# 1,10-Phenanthroline-5,6-dione ethanol monosolvate

**DOI:** 10.1107/S1600536814008241

**Published:** 2014-04-18

**Authors:** Jing-Wei Dai, Zhao-Yang Li, Osamu Sato

**Affiliations:** a Institute for Materials Chemistry and Engineering, Kyushu University, 6-1 Kasuga-koen, Kasuga, Fukuoka 816-8580, Japan; b Department of Chemistry, Graduate School of Science, Tohoku University, 6-3 Aramaki-Aza-Aoba, Aoba-ku, Sendai 980-8578, Japan

## Abstract

In the title compound, C_12_H_6_N_2_O_2_·C_2_H_5_OH, the mol­ecule of the 1,10-phenanthroline-5,6-dione is approximately planar, with a maximum deviation of 0.051 (1) Å. In the crystal, mol­ecules are linked by O—H⋯N and weak C—H⋯O hydrogen bonds, forming supra­molecular chains propagating along [110]. π–π stacking inter­actions are observed between the pyridine rings of neighbouring chains, the centroid–centroid separations being 3.6226 (11) and 3.7543 (11) Å.

## Related literature   

For background to and applications of 1,10-phenanthroline-5,6-dione, see: Smith & Cagle (1947 ▶); Ma *et al.* (2010 ▶); Goss & Abruna (1985 ▶); Murphy *et al.* (2011 ▶); Wu *et al.* (1996 ▶); Pinczewska *et al.* (2012 ▶); Poteet & MacDonnell (2013 ▶); Wu *et al.* (2002 ▶); Poteet *et al.* 2013 ▶); Paw *et al.* (1998 ▶). For the synthesis, see: Paw & Eisenberg (1997 ▶). For a related structure, see: Calderazzo *et al.* (1999 ▶).
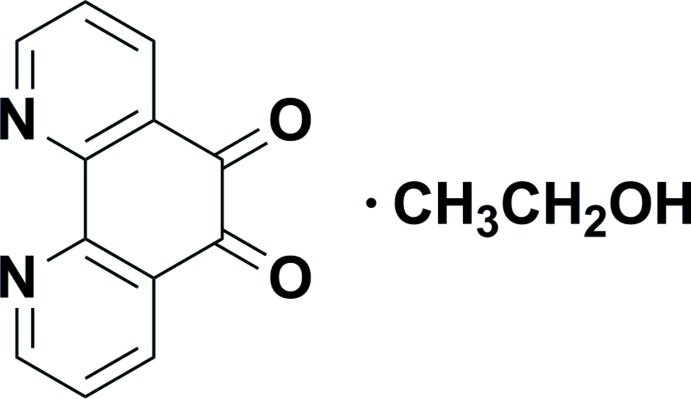


## Experimental   

### 

#### Crystal data  

C_12_H_6_N_2_O_2_·C_2_H_6_O
*M* *_r_* = 256.26
Triclinic, 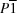

*a* = 7.3064 (15) Å
*b* = 9.1055 (18) Å
*c* = 9.7291 (19) Å
α = 96.47 (3)°
β = 101.68 (3)°
γ = 109.83 (3)°
*V* = 584.6 (2) Å^3^
*Z* = 2
Mo *K*α radiation
μ = 0.10 mm^−1^
*T* = 123 K
0.20 × 0.20 × 0.20 mm

#### Data collection  

Rigaku Saturn724+ diffractometer
5059 measured reflections
2252 independent reflections
2074 reflections with *I* > 2σ(*I*)
*R*_int_ = 0.020

#### Refinement  

*R*[*F* ^2^ > 2σ(*F* ^2^)] = 0.040
*wR*(*F* ^2^) = 0.114
*S* = 1.06
2252 reflections
185 parameters
1 restraint
H atoms treated by a mixture of independent and constrained refinement
Δρ_max_ = 0.24 e Å^−3^
Δρ_min_ = −0.17 e Å^−3^

### 

Data collection: *CrystalClear* (Rigaku, 2008 ▶); cell refinement: *CrystalClear*; data reduction: *CrystalClear*; program(s) used to solve structure: *SHELXS97* (Sheldrick, 2008 ▶); program(s) used to refine structure: *SHELXL97* (Sheldrick, 2008 ▶); molecular graphics: *ORTEP-3 for Windows* (Farrugia, 2012 ▶) and *Mercury* (Macrae *et al.*, 2008 ▶); software used to prepare material for publication: *publCIF* (Westrip, 2010 ▶).

## Supplementary Material

Crystal structure: contains datablock(s) I, global. DOI: 10.1107/S1600536814008241/xu5783sup1.cif

Structure factors: contains datablock(s) I. DOI: 10.1107/S1600536814008241/xu5783Isup2.hkl

Supporting information file. DOI: 10.1107/S1600536814008241/xu5783Isup3.cml

CCDC reference: 996896

Additional supporting information:  crystallographic information; 3D view; checkCIF report

## Figures and Tables

**Table 1:** Hydrogen-bond geometry (Å, °)

*D*—H⋯*A*	*D*—H	H⋯*A*	*D*⋯*A*	*D*—H⋯*A*
O3—H3*O*⋯N1^i^	0.85 (1)	2.08 (1)	2.8258 (19)	146 (2)
C1—H1⋯O2^ii^	0.95	2.53	3.3381 (19)	143

Symmetry codes: (i) 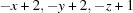; (ii) 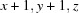.

**Fig. 1.**
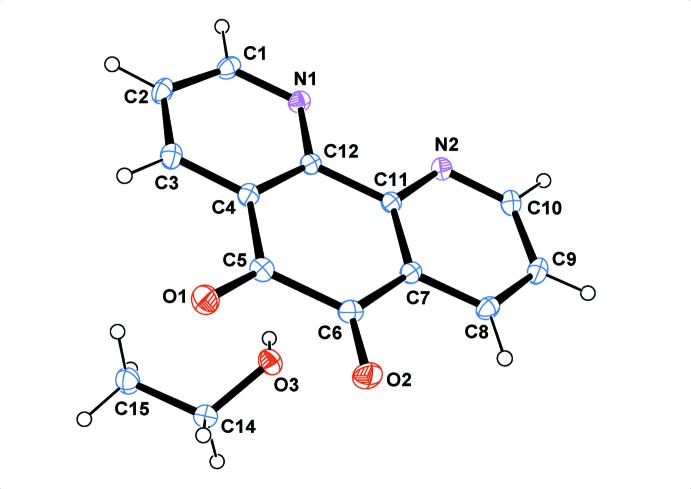
Crystal structure of the title compound with labeling and displacement ellipsoids drawn at the 30% probability level.

**Fig. 2.**
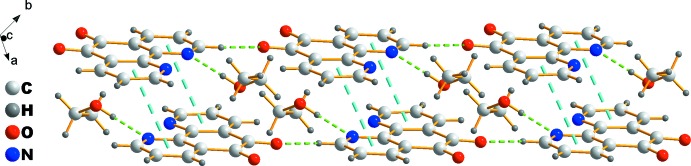
Crystal packing of the title compound. Intermolecular O—H···N and C—H···O hydrogen bonds are shown as green dashed lines, and π-π stacking interactions between molecules are shown as blue dashed lines.

## supplementary crystallographic information

### 2.1. Synthesis and crystallization

The title compound was prepared according to literature method (Paw & Eisenberg, 1997). An ice-cold mixture of concentrated H_2_SO_4_ (40 mL) and HNO_3_ (20 mL) was added to 4 g of 1,10-phenanthroline (0.02 mol) and 4 g of KBr (0.03 mol). The mixture was heated at 90 ^o^C for 3 h. The hot yellow solution was poured over 200 mL of ice and neutralized carefully with sodium hydroxide until neutral to slightly acidic pH. Extraction with CH_2_Cl_2_ (4*100 mL) followed by drying with Na_2_SO_4_ and removal of solvent gave 2.8 g (yield = 67%) of 1,10-phenanthroline-5,6-dione. This product was purified further by crystallization from ethanol.

### 2.2. Refinement

Crystal data, data collection and structure refinement details are summarized in Table 1. Carbon-bound H-atoms were placed in calculated positions and were included in the refinement in the riding model approximation with U_iso_(H) = 1.5U_eq_(C) for methyl H atoms and 1.2U_eq_(C) for the others. The hy­droxy H atom was located in a difference Fourier map, and was refined with distance restraints of O—H = 0.84±0.01, U_iso_(H) = 1.2U_eq_(O).

### 3. Results and discussion

1,10-Phenanthroline-5,6-dione has been known for many years (Smith & Cagle, 1947), and its chelating ability as either a di­imine or a catecholate was important in coordination chemistry (Ma *et al.*, 2010, Goss & Abruna, 1985, Murphy *et al.*, 2011), analytical chemistry (Wu *et al.*, 1996, Pinczewska *et al.*, 2012) and biophysical chemistry (Poteet & MacDonnell, 2013, Wu *et al.*, 2002, Poteet *et al.*, 2013). Moreover, it can become as the bridging ligand, which has shown very inter­esting function in multinuclear complexes (Paw *et al.*, 1998, Paw & Eisenberg, 1997, Calderazzo *et al.*, 1999).

According to the structural analysis, the bond lengths and angles of the title compound are generally within normal ranges. The asymmetric unit of the title compound consists of one 1,10-phenanthroline-5,6-dione molecule and one ethanol molecule. Between molecules, O—H···N and C—H···O hydrogen bonds can be found that further form one-dimensional chain. The weak π···π stacking inter­actions between adjacent chains are also observed [centroid– centroid separations being 3.6226 (11) and 3.7543 (11) Å].

### Crystal data

**Table:**

C_12_H_6_N_2_O_2_·C_2_H_6_O	*Z* = 2
*M**_r_* = 256.26	*F*(000) = 268
Triclinic, *P*1	*D*_x_ = 1.456 Mg m^−^^3^
Hall symbol: -P 1	Mo *K*α radiation, λ = 0.71073 Å
*a* = 7.3064 (15) Å	Cell parameters from 2355 reflections
*b* = 9.1055 (18) Å	θ = 3.1–30.2°
*c* = 9.7291 (19) Å	µ = 0.10 mm^−^^1^
α = 96.47 (3)°	*T* = 123 K
β = 101.68 (3)°	Block, yellow
γ = 109.83 (3)°	0.20 × 0.20 × 0.20 mm
*V* = 584.6 (2) Å^3^	

### Data collection

**Table:**

Rigaku Saturn724+ diffractometer	2074 reflections with *I* > 2σ(*I*)
Radiation source: Rotating Anode	*R*_int_ = 0.020
Confocal monochromator	θ_max_ = 26.0°, θ_min_ = 3.1°
Detector resolution: 28.5714 pixels mm^-1^	*h* = −9→9
ω scans	*k* = −11→11
5059 measured reflections	*l* = −11→11
2252 independent reflections	

### Refinement

**Table:**

Refinement on *F*^2^	Primary atom site location: structure-invariant direct methods
Least-squares matrix: full	Secondary atom site location: difference Fourier map
*R*[*F*^2^ > 2σ(*F*^2^)] = 0.040	Hydrogen site location: inferred from neighbouring sites
*wR*(*F*^2^) = 0.114	H atoms treated by a mixture of independent and constrained refinement
*S* = 1.06	*w* = 1/[σ^2^(*F*_o_^2^) + (0.0709*P*)^2^ + 0.118*P*] where *P* = (*F*_o_^2^ + 2*F*_c_^2^)/3
2252 reflections	(Δ/σ)_max_ < 0.001
185 parameters	Δρ_max_ = 0.24 e Å^−^^3^
1 restraint	Δρ_min_ = −0.17 e Å^−^^3^

### Special details

**Table:**

Geometry. All e.s.d.'s (except the e.s.d. in the dihedral angle between two l.s. planes) are estimated using the full covariance matrix. The cell e.s.d.'s are taken into account individually in the estimation of e.s.d.'s in distances, angles and torsion angles; correlations between e.s.d.'s in cell parameters are only used when they are defined by crystal symmetry. An approximate (isotropic) treatment of cell e.s.d.'s is used for estimating e.s.d.'s involving l.s. planes.
Refinement. Refinement of *F*^2^ against ALL reflections. The weighted *R*-factor *wR* and goodness of fit *S* are based on *F*^2^, conventional *R*-factors *R* are based on *F*, with *F* set to zero for negative *F*^2^. The threshold expression of *F*^2^ > σ(*F*^2^) is used only for calculating *R*-factors(gt) *etc*. and is not relevant to the choice of reflections for refinement. *R*-factors based on *F*^2^ are statistically about twice as large as those based on *F*, and *R*- factors based on ALL data will be even larger.

### Fractional atomic coordinates and isotropic or equivalent isotropic displacement parameters (Å^2^)

**Table:**

	*x*	*y*	*z*	*U*_iso_*/*U*_eq_	
C4	0.66468 (17)	0.96363 (14)	0.27101 (12)	0.0211 (3)	
C11	0.69855 (17)	0.93910 (13)	0.52865 (12)	0.0196 (3)	
C5	0.50156 (18)	0.80403 (14)	0.22423 (12)	0.0237 (3)	
C7	0.54977 (17)	0.78514 (14)	0.49173 (12)	0.0211 (3)	
C6	0.43997 (17)	0.70995 (13)	0.34035 (13)	0.0239 (3)	
C12	0.75757 (16)	1.02974 (13)	0.41609 (12)	0.0200 (3)	
C8	0.50365 (18)	0.70329 (14)	0.60099 (13)	0.0250 (3)	
H8	0.4042	0.5987	0.5794	0.030*	
C9	0.60544 (18)	0.77748 (15)	0.74108 (13)	0.0270 (3)	
H9	0.5787	0.7248	0.8179	0.032*	
C3	0.72616 (18)	1.05260 (15)	0.16952 (13)	0.0252 (3)	
H3	0.6651	1.0108	0.0704	0.030*	
C1	0.95979 (18)	1.25750 (14)	0.36088 (14)	0.0262 (3)	
H1	1.0632	1.3603	0.3919	0.031*	
C10	0.74812 (18)	0.93131 (15)	0.76688 (13)	0.0261 (3)	
H10	0.8166	0.9818	0.8636	0.031*	
C2	0.87625 (19)	1.20164 (15)	0.21448 (14)	0.0272 (3)	
H2	0.9213	1.2644	0.1475	0.033*	
O1	0.41332 (14)	0.74720 (11)	0.09901 (9)	0.0331 (3)	
O2	0.30275 (14)	0.58111 (10)	0.30567 (10)	0.0333 (3)	
N2	0.79532 (15)	1.01274 (12)	0.66490 (10)	0.0237 (2)	
N1	0.90359 (15)	1.17542 (12)	0.46054 (11)	0.0233 (2)	
C15	0.8686 (2)	0.65765 (16)	0.07549 (14)	0.0336 (3)	
H15A	0.8325	0.7503	0.0621	0.050*	
H15B	0.8372	0.5885	−0.0177	0.050*	
H15C	1.0130	0.6934	0.1211	0.050*	
C14	0.7508 (2)	0.56671 (15)	0.16901 (14)	0.0312 (3)	
O3	0.76490 (13)	0.66129 (10)	0.30034 (9)	0.0291 (2)	
H14A	0.598 (3)	0.5214 (19)	0.1167 (17)	0.042 (4)*	
H14B	0.797 (3)	0.479 (2)	0.1910 (18)	0.048 (5)*	
H3O	0.8864 (17)	0.719 (2)	0.3431 (19)	0.058 (5)*	

### Atomic displacement parameters (Å^2^)

**Table:**

	*U*^11^	*U*^22^	*U*^33^	*U*^12^	*U*^13^	*U*^23^
C4	0.0190 (6)	0.0245 (6)	0.0236 (6)	0.0108 (5)	0.0073 (4)	0.0073 (5)
C11	0.0173 (5)	0.0214 (6)	0.0226 (6)	0.0091 (4)	0.0069 (4)	0.0051 (4)
C5	0.0215 (6)	0.0259 (6)	0.0238 (6)	0.0102 (5)	0.0044 (5)	0.0036 (5)
C7	0.0188 (6)	0.0219 (6)	0.0252 (6)	0.0091 (5)	0.0077 (4)	0.0064 (4)
C6	0.0205 (6)	0.0222 (6)	0.0291 (6)	0.0078 (5)	0.0068 (5)	0.0043 (5)
C12	0.0168 (5)	0.0216 (6)	0.0238 (6)	0.0086 (4)	0.0070 (4)	0.0053 (4)
C8	0.0217 (6)	0.0231 (6)	0.0330 (7)	0.0085 (5)	0.0107 (5)	0.0100 (5)
C9	0.0266 (6)	0.0333 (7)	0.0277 (6)	0.0138 (5)	0.0118 (5)	0.0145 (5)
C3	0.0242 (6)	0.0326 (7)	0.0240 (6)	0.0143 (5)	0.0085 (5)	0.0095 (5)
C1	0.0215 (6)	0.0234 (6)	0.0355 (7)	0.0065 (5)	0.0113 (5)	0.0107 (5)
C10	0.0260 (6)	0.0332 (7)	0.0215 (6)	0.0124 (5)	0.0077 (5)	0.0069 (5)
C2	0.0268 (6)	0.0324 (7)	0.0318 (7)	0.0150 (5)	0.0157 (5)	0.0163 (5)
O1	0.0338 (5)	0.0333 (5)	0.0238 (5)	0.0072 (4)	0.0005 (4)	0.0019 (4)
O2	0.0290 (5)	0.0249 (5)	0.0346 (5)	−0.0012 (4)	0.0051 (4)	0.0027 (4)
N2	0.0234 (5)	0.0257 (5)	0.0222 (5)	0.0088 (4)	0.0070 (4)	0.0044 (4)
N1	0.0200 (5)	0.0223 (5)	0.0276 (5)	0.0068 (4)	0.0076 (4)	0.0060 (4)
C15	0.0321 (7)	0.0332 (7)	0.0282 (7)	0.0040 (5)	0.0083 (5)	0.0019 (5)
C14	0.0388 (8)	0.0250 (6)	0.0296 (7)	0.0104 (5)	0.0116 (6)	0.0043 (5)
O3	0.0255 (5)	0.0329 (5)	0.0261 (5)	0.0092 (4)	0.0059 (4)	0.0007 (4)

### Geometric parameters (Å, º)

**Table:**

C4—C3	1.3950 (17)	C3—C2	1.3776 (19)
C4—C12	1.3995 (17)	C3—H3	0.9500
C4—C5	1.4818 (18)	C1—N1	1.3355 (16)
C11—N2	1.3436 (16)	C1—C2	1.3907 (18)
C11—C7	1.4037 (17)	C1—H1	0.9500
C11—C12	1.4899 (16)	C10—N2	1.3357 (16)
C5—O1	1.2171 (15)	C10—H10	0.9500
C5—C6	1.5411 (17)	C2—H2	0.9500
C7—C8	1.3972 (17)	C15—C14	1.5026 (18)
C7—C6	1.4843 (18)	C15—H15A	0.9800
C6—O2	1.2128 (16)	C15—H15B	0.9800
C12—N1	1.3448 (16)	C15—H15C	0.9800
C8—C9	1.3813 (18)	C14—O3	1.4225 (15)
C8—H8	0.9500	C14—H14A	1.040 (18)
C9—C10	1.3914 (19)	C14—H14B	0.995 (18)
C9—H9	0.9500	O3—H3O	0.850 (10)
			
C3—C4—C12	118.58 (11)	C4—C3—H3	120.3
C3—C4—C5	119.93 (11)	N1—C1—C2	124.01 (11)
C12—C4—C5	121.48 (11)	N1—C1—H1	118.0
N2—C11—C7	122.79 (11)	C2—C1—H1	118.0
N2—C11—C12	116.36 (10)	N2—C10—C9	124.42 (12)
C7—C11—C12	120.85 (11)	N2—C10—H10	117.8
O1—C5—C4	122.47 (12)	C9—C10—H10	117.8
O1—C5—C6	119.54 (11)	C3—C2—C1	117.99 (11)
C4—C5—C6	117.97 (10)	C3—C2—H2	121.0
C8—C7—C11	118.69 (11)	C1—C2—H2	121.0
C8—C7—C6	119.96 (11)	C10—N2—C11	117.08 (11)
C11—C7—C6	121.35 (11)	C1—N1—C12	117.77 (11)
O2—C6—C7	122.90 (12)	C14—C15—H15A	109.5
O2—C6—C5	119.48 (11)	C14—C15—H15B	109.5
C7—C6—C5	117.60 (10)	H15A—C15—H15B	109.5
N1—C12—C4	122.28 (11)	C14—C15—H15C	109.5
N1—C12—C11	117.04 (10)	H15A—C15—H15C	109.5
C4—C12—C11	120.68 (11)	H15B—C15—H15C	109.5
C9—C8—C7	118.69 (11)	O3—C14—C15	114.19 (11)
C9—C8—H8	120.7	O3—C14—H14A	104.6 (9)
C7—C8—H8	120.7	C15—C14—H14A	109.3 (9)
C8—C9—C10	118.31 (11)	O3—C14—H14B	108.5 (10)
C8—C9—H9	120.8	C15—C14—H14B	109.8 (10)
C10—C9—H9	120.8	H14A—C14—H14B	110.5 (14)
C2—C3—C4	119.37 (12)	C14—O3—H3O	111.5 (14)
C2—C3—H3	120.3		

### Hydrogen-bond geometry (Å, º)

**Table:**

*D*—H···*A*	*D*—H	H···*A*	*D*···*A*	*D*—H···*A*
O3—H3*O*···N1^i^	0.85 (1)	2.08 (1)	2.8258 (19)	146 (2)
C1—H1···O2^ii^	0.95	2.53	3.3381 (19)	143

Symmetry codes: (i) −*x*+2, −*y*+2, −*z*+1; (ii) *x*+1, *y*+1, *z*.

## References

[bb1] Calderazzo, F., Marchetti, F., Pampaloni, G. & Passarelli, V. (1999). *J. Chem. Soc. Dalton Trans.* pp. 4389–4396.

[bb2] Farrugia, L. J. (2012). *J. Appl. Cryst.* **45**, 849–854.

[bb3] Goss, C. A. & Abruna, H. D. (1985). *Inorg. Chem.* **24**, 4263–4267.

[bb4] Ma, Q., Zhu, M. L., Yuan, C. X., Feng, S. S., Lu, L. P. & Wang, Q. M. (2010). *Cryst. Growth Des.* **10**, 1706–1714.

[bb5] Macrae, C. F., Bruno, I. J., Chisholm, J. A., Edgington, P. R., McCabe, P., Pidcock, E., Rodriguez-Monge, L., Taylor, R., van de Streek, J. & Wood, P. A. (2008). *J. Appl. Cryst.* **41**, 466–470.

[bb6] Murphy, D. M., McNamara, K., Richardson, P., Sanchez-Romaguera, V., Winpenny, R. E. P. & Yellowlees, L. J. (2011). *Inorg. Chim. Acta*, **374**, 435–441.

[bb7] Paw, W., Connick, W. B. & Eisenberg, R. (1998). *Inorg. Chem.* **37**, 3919–3926. 10.1021/ic971447k 11670504

[bb8] Paw, W. & Eisenberg, R. (1997). *Inorg. Chem.* **36**, 2287–2293. 10.1021/ic9610851 11669862

[bb9] Pinczewska, A., Sosna, M., Bloodworth, S., Kilburn, J. D. & Bartlett, P. N. (2012). *J. Am. Chem. Soc.* **134**, 18022–18033. 10.1021/ja307390x 23046387

[bb10] Poteet, S. A. & MacDonnell, F. M. (2013). *Dalton Trans.* **42**, 13305–13307. 10.1039/c3dt51664d 23925310

[bb11] Poteet, S. A., Majewski, M. B., Breitbach, Z. S., Griffith, C. A., Singh, S., Armstrong, D. W., Wolf, M. O. & MacDonnell, F. M. (2013). *J. Am. Chem. Soc.* **135**, 2419–2422. 10.1021/ja3106863 23350926

[bb12] Rigaku (2008). *CrystalClear* Rigaku Corporation, Tokyo, Japan.

[bb13] Sheldrick, G. M. (2008). *Acta Cryst.* A**64**, 112–122. 10.1107/S0108767307043930 18156677

[bb14] Smith, G. F. & Cagle, F. W. (1947). *J. Org. Chem.* **12**, 781–784. 10.1021/jo01170a007 18919731

[bb15] Westrip, S. P. (2010). *J. Appl. Cryst.* **43**, 920–925.

[bb16] Wu, J. Z., Li, H., Zhang, J. G. & Xu, J. H. (2002). *Inorg. Chem. Commun.* **5**, 71–75.

[bb17] Wu, Q., Maskus, M., Pariente, F., Tobalina, F., Fernandez, V. M., Lorenzo, E. & Abruna, H. D. (1996). *Anal. Chem.* **68**, 3688–3696.

